# Using the 6 minute walk to investigate endurance in patients with HTLV associated myelopathy

**DOI:** 10.1186/1742-4690-11-S1-P20

**Published:** 2014-01-07

**Authors:** A Adonis, G Taylor

**Affiliations:** 1 National Centre For Human Retrovirology (NCHR), St Mary’s Hospital, Imperial College Healthcare NHS Trust, London, UK; 2 Imperial College London, London, UK

## 

Patients with HTLV-Associated-Myelopathy (pwHAM) predominantly have proximal leg weakness, and reduced activities of daily living (ADLs). 92% of pwHAM attending NCHR use a walking aid and experience difficulty with walking long distances, thus many are housebound, or community ambulators. The 6 minute walk test (6MWT) measures walking endurance and was measured at 2 timepoints (T1 & T2), 1 year(yr) apart. We retrospectively analysed 36 patients’ notes (26♀: 10♂; mean age 56.8yrs; mean duration of HAM 10.7yrs). We correlated, regressed and t-tested 10m timed walk (10mTW), walking aid used, pain scores, distance covered, time taken and velocity. Significant differences were found between T1&T2 for: 6MWTime p=0.02; 6MWVelocity p=0.04 & 10mTW p=0.04. 6MW is reliable (ICC: 0.83).10mTW inversely correlated with the 6MWTDistance covered T1p=0.002 & T2 p=0.022 & 6MWTVelocity T1p=0.000 & T2 p=0.002.10mTW at T1 predicted 6MWDistance (p=0.004), T1 6MWVelocity predicted T2 distance walked. Walking aids predicted 10mTW time (p=0.00 at T1 & T2); 6 MWDistance covered (T1=p=0.002;T2:p=0.00) and velocity (T1&T2:p=0.00). Duration of disease (p=0.34), interval between tests (p=0.57) & age (p=0.75) did not predict the 6 MWT (distance or time) or the 10mTW. Average pain score changed 8.3% between T1&T2 (p=0.75). No element of the 6 MW test or the 10mTW correlated with pain at either time point. Walking endurance is an important component of walking capacity. The 6MWT appears to be a reliable measure to use in patients with HAM. The results demonstrate that pwHAM are more dependent in their ADLs, are a falls risk, walk limited distances & have limited endurance.

